# In Vitro Evaluation of the Anti-Inflammatory Effect of KMUP-1 and In Vivo Analysis of Its Therapeutic Potential in Osteoarthritis

**DOI:** 10.3390/biomedicines9060615

**Published:** 2021-05-28

**Authors:** Shang-En Huang, Erna Sulistyowati, Yu-Ying Chao, Bin-Nan Wu, Zen-Kong Dai, Jong-Hau Hsu, Jwu-Lai Yeh

**Affiliations:** 1 Graduate Institute of Medicine, College of Medicine, Kaohsiung Medical University, Kaohsiung 80708, Taiwan; eva_1433@yahoo.com.tw (S.-E.H.); dr_erna@unisma.ac.id (E.S.); binnan@kmu.edu.tw (B.-N.W.); zenkong@kmu.edu.tw (Z.-K.D.); 2 Faculty of Medicine, University of Islam Malang, Malang 65146, Indonesia; 3 Department of Public Health, College of Health Sciences, Kaohsiung Medical University, Kaohsiung 80708, Taiwan; yuyich@kmu.edu.tw; 4 Department of Pharmacology, College of Medicine, Kaohsiung Medical University, Kaohsiung 80708, Taiwan; 5 Department of Pediatrics, Kaohsiung Medical University Hospital, Kaohsiung 80708, Taiwan; 6 Department of Pediatrics, College of Medicine, Kaohsiung Medical University, Kaohsiung 80708, Taiwan; 7 Department of Medical Research, Kaohsiung Medical University Hospital, Kaohsiung 80708, Taiwan; 8 Department of Marine Biotechnology and Resources, National Sun Yat-sen University, Kaohsiung 80424, Taiwan

**Keywords:** xanthine-derived KMUP-1, anti-inflammation, NF-κB, SIRT1, osteoarthritis

## Abstract

Osteoarthritis is a degenerative arthropathy that is mainly characterized by dysregulation of inflammatory responses. KMUP-1, a derived chemical synthetic of xanthine, has been shown to have anti-inflammatory and antioxidant properties. Here, we aimed to investigate the in vitro anti-inflammatory and in vivo anti-osteoarthritis effects of KMUP-1. Protein and gene expressions of inflammation markers were determined by ELISA, Western blotting and microarray, respectively. RAW264.7 mouse macrophages were cultured and pretreated with KMUP-1 (1, 5, 10 μM). The productions of TNF-α, IL-6, MMP-2 and MMP- 9 were reduced by KMUP-1 pretreatment in LPS-induced inflammation of RAW264.7 cells. The expressions of iNOS, TNF-α, COX-2, MMP-2 and MMP-9 were also inhibited by KMUP-1 pretreatment. The gene expression levels of TNF and COX families were also downregulated. In addition, KMUP-1 suppressed the activations of ERK, JNK and p38 as well as phosphorylation of IκBα/NF-κB signaling pathways. Furthermore, SIRT1 inhibitor attenuated the inhibitory effect of KMUP-1 in LPS-induced NF-κB activation. In vivo study showed that KMUP-1 reduced mechanical hyperalgesia in monoiodoacetic acid (MIA)-induced rats OA. Additionally, KMUP-1 pretreatment reduced the serum levels of TNF-α and IL-6 in MIA-injected rats. Moreover, macroscopic and histological observation showed that KMUP-1 reduced articular cartilage erosion in rats. Our results demonstrated that KMUP-1 inhibited the inflammatory responses and restored SIRT1 in vitro, alleviated joint-related pain and cartilage destruction in vivo. Taken together, KMUP-1 has the potential to improve MIA-induced articular cartilage degradation by inhibiting the levels and expression of inflammatory mediators suggesting that KMUP-1 might be a potential therapeutic agent for OA.

## 1. Introduction

Osteoarthritis (OA) has long been defined as a degenerative disease characterized by continuously articular cartilage damage, formation of osteophyte, and subchondral bones alteration, resulting in devastating chronic pain in affected individuals [1]. Inflammatory cytokines have a primarily destructive impact on articular cartilage. It is a multilevel im-pact that involves not only the induction of aging and apoptosis of chondrocytes, but also a decrease in the synthesis of the key components of extracellular matrix, such as proteo-glycans, and type II collagen [2]. Tumor necrosis factor-α (TNF-α) and interleukin-1β (IL-1β) are predominant inflammatory cytokines that are believed to be involved in the progression of OA [3]. Furthermore, inflammation in the articular tissue is directly related with cartilage degradation that increases mediators and molecules including inducible nitric oxide synthase (iNOS), IL-8, and IL-6. Initiation of the NF-κB signaling increases the production of nitric oxide (NO), cyclooxygenase-2 (COX-2) and matrix metalloproteinases (MMPs), which account for the articular cartilage breakdown [4,5,6]. There are several factors such as aging, genetic, and mechanical-associated factors that are involved in the pathogenesis of OA. Eventually, these factors lead to synovitis, apoptosis, and cartilage destruction [4].

The greatest risk factor for OA is advanced age and it is undoubtful whether cell senescence and aging contribute in the joint tissues’ alterations during the development of OA. The silent information regulator 2 type 1 (also known as sirtuin 1 (SIRT1) is a member of sirtuin family proteins, a popular group of antiaging genes [7]. A variety of age-related diseases such as cancer, type 2 diabetes, cardiovascular disease, Alzheimer’s disease, arthritis, osteoporosis, as well as OA are associated with SIRT1 [8]. Hence, there has been a significant increase in new investigations that aim to elucidate the mechanisms of sirtuin function and their roles in cartilage biology, skeletal development, and pathologies such as OA, rheumatoid arthritis (RA), and intervertebral disc degeneration (IVD) [9]. In cartilage homeostasis, the expression of SIRT 1 protein is important which prevents cell death and promotes cell survival through an enzymatically independent mechanism. Cartilage destruction in OA is thought to be mediated by two main enzyme families: the MMP and ADAMTS (A Disintegrin and Metalloproteinase with Thrombospondin motifs) enzymes are responsible for cartilage collagen breakdown [2]. It is confirmed that SIRT1 reveals anticatabolic and anti-inflammatory effects in OA. A plethora of recent studies have confirmed that SIRT1 indeed inhibited the NF-κB signaling, and the activation of SIRT1 could alleviate a multitude of NF-κB-driven inflammatory and metabolic disorders [10,11]. This implies that SIRT1 activators could exert significant benefits in the treatment of OA [9]. For that reason, finding drugs that prevent proinflammatory cytokines would be beneficial to suppress inflammation, which may assuredly contribute for OA therapeutic strategies.

**Figure 1:**
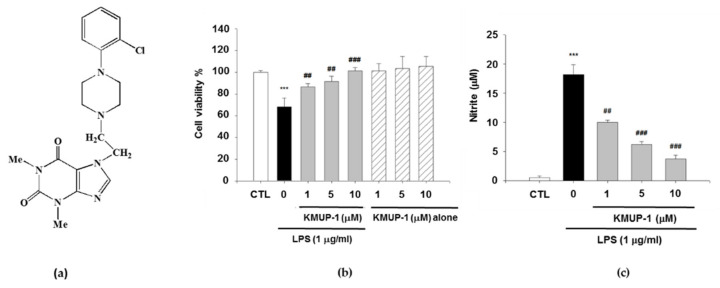
Effects of KMUP-1 on cell viability and NO production in LPS-induced RAW 264.7 cells. (**a**) The chemical structure of KMUP-1. (**b**) The cell viability was determined by MTT assay. (**c**) Griess assay was used to identify nitrite content in the culture media. KMUP-1 treatment was 1 h administered prior to 24 h LPS-induced inflammation exposure. Values are presented as mean ± SEM, *n* = 6. *** *p* < 0.001 compared to control group (CTL). ## *p* < 0.01 and ### *p* < 0.001 compared to LPS group.

KMUP-1, a chemical synthetic xanthine-based derivative (Figure 1a) has been shown to possess multifunctional properties, including anti-inflammatory, cardioprotective, and neuroprotective roles [12,13,14]. Our recent study has shown that KMUP-1 suppresses RANKL-induced osteoclastogenesis and ovariectomy-induced bone loss [15]. Additionally, KMUP-1 stimulates osteoblast differentiation [16]. In this study, we investigated the in vitro anti-inflammatory and in vivo anti-osteoarthritis effects of KMUP-1.

## 2. Materials and Methods

### 2.1. Materials and Reagents

KMUP-1 hydrochloride (KMUP-1) was synthesized in our laboratory [9]. KMUP-1 was dissolved in distilled water for experiments. Thiazolyl Blue Tetrazolium Bromide (MTT) powder from Sigma-Aldrich Inc. (St. Louis, MO, USA). Enzyme-linked immunosorbent assay (ELISA) kits were purchased from R&D Systems (Minneapolis, MN, USA). The ELISA kit consisted of mouse TNF-α (DY410), mouse IL-6 (DY406), mouse MMP-2 (MMP200), mouse MMP-9 (DY6718), rat TNF-α (DY510), and rat IL-6 (DY506). SIRT1 inhibitor (Ex-527) was purchased from Santa Cruz Biotechnology (Santa Cruz, CA, USA). The secondary antibodies were from Merck Millipore. Other reagents were purchased from Sigma-Aldrich. The GIBCO BRL Life Technologies (Grand Island, NY, USA) provided Dulbecco’s modified Eagle’s medium (DMEM), fetal bovine serum (FBS), streptomycin, penicillin, and all other tissue culture reagents.

### 2.2. Cell Culture and Lipopolysaccharide-Induced Inflammation

The RAW264.7 mouse cell line was obtained from the Bioresource Collection and Research Center in Taiwan. Cells were maintained in DMEM medium supplemented with 10% FBS, 2 mM glutamine, 100 U/mL penicillin at 37 °C and in a humidified 5% CO_2_. After KMUP-1 pretreatment, the cells were incubated with 1 µg/mL lipopolysaccharides (LPS) for 24 h to stimulate inflammation.

### 2.3. Cell Viability Assay

RAW264.7 cells were cultured in 24-well plates followed by 1 h KMUP-1 pretreatment at various concentrations and then LPS incubation for 24 h. MTT solution (0.5 mg/mL) was added and incubated for 4 h at 37 °C. After MTT solution was removed, isopropanol was then added and the cells were shaken for 10 min. The MTT formazan crystals were quantified by determining the absorbance at 540 and 630 nm, using an enzyme-linked immunosorbent assay (ELISA) reader (DYNEX Technologies, Denkendorf, Germany).

### 2.4. Measurement of Nitrite Oxide

To determine nitrite oxide (NO) generation, we measured the accumulation of nitrite, an NO metabolite. This indirect indicator of NO production was assayed in the cell culture medium using Griess reagent (1% sulfanilamide and 0.1% N-(1-naphthyl) ethylenediamide in 5% phosphoric acid). The cells culture media were collected and incubated with an equal volume of Griess reagent for 10 min at room temperature. The absorbance was measured at 540 nm with ELISA reader (DYNEX Technologies, Denkendorf, Germany).

### 2.5. Measurement of TNF-α, IL-6, MMP-2, and MMP-9

The RAW 264.7 cells were pretreated with KMUP-1 for 1 h and then were induced inflammation with LPS. To measure TNF-α, IL-6, MMP-2, and MMP-9, the cells culture media were collected. The cytokines production of TNF-α, IL-6, MMP-2, and MMP-9 were measured using ELISA kit according to the manufacturer’s protocol (R&D Systems, Minneapolis, MN, USA).

### 2.6. Western Blot Analysis

The cells were treated with indicated concentrations of KMUP-1 and LPS-stimulated inflammation. The reactions were terminated by washing with PBS and then cells were harvested. Total cell extracts were prepared in lysis buffer 20 mM Tris–HCl (pH 7.5), 1 mM dithiothreitol (DTT), 5 mM EGTA, 2 mM EDTA, 0.5 mM PMSF, 20 μM leupeptin, and 20 μM aprotinin. The cell lysate was centrifuged at 12,000× *g* for 20 min, and the supernatant fraction was collected for Western blot. Cell extracts (30 μg/mL of protein homogenate) were diluted in 5× Sample buffer (Biomate, Tapei, Taiwan). An equivalent amount of protein was resolved by SDS-polyacrylamide gel electrophoresis (PAGE) (10–12%) and transferred to polyvinylidene difluoride (PVDF) membranes. The membranes were blocked with a blocking buffer (5% non-fat dry milk in Tris-buffered saline) for 1 h. Subsequently, all of it was covered up with related primary antibodies overnight, respectively. The primary antibodies we used in the study were rabbit polyclonal anti-iNOS (ab15323, abcam; 1;1000), rabbit polyclonal anti-TNF alpha (ab6671, abcam; 1;1000), rabbit polyclonal anti-COX2 (ab15191, abcam; 1;1000), mouse monoclonal anti-MMP-2 (MA5-13590, Thermo; 1;1000), mouse monoclonal anti-MMP-9 (MA5-14228, Thermo; 1;1000), mouse monoclonal anti-beta Actin (GT629630, GeneTex; 1;1000), rabbit polyclonal anti-phospho ERK1/2 (Thr202/Tyr204, #9101, Cell signaling; 1;1000), rabbit polyclonal anti-ERK1/2 (#9102, Cell signaling; 1;1000), rabbit monoclonal anti-phospho JNK (Thr183/Tyr185, #4671, Cell signaling; 1;1000), rabbit polyclonal anti-JNK (#9252, Cell signaling; 1;1000), rabbit polyclonal anti-phospho p38 (Thr180/Tyr182, #4511, Cell signaling; 1;1000), rabbit polyclonal anti-p38 (#9212, Cell signaling; 1;1000), mouse monoclonal anti-phospho-I*κ*Bα (Ser32/36, #9246, Cell signaling; 1;1000), rabbit monoclonal anti-I*κ*Bα (44D4, #4812, Cell signaling; 1;1000), rabbit polyclonal anti-NF-κB p65 (GT107678, GeneTex; 1;1000), rabbit monoclonal anti-phospho-NF-κB p65 (Ser536, #3033, Cell signaling; 1;1000), mouse monoclonal anti-SIRT1 (ab110304, abcam; 1;1000). Afterward, it was overlaid in appropriate horseradish peroxidase-linked secondary antibody (1;1000) for 1 h, and the expression level of proteins were detected with enhanced chemiluminescence reagents (Merck Millipore, Burlington, MA, USA).

### 2.7. Microarray

Genome-wide expression analysis was conducted using RNA isolated from RAW 264.7 mouse cells (Mouse OneArray Plus, Phalanxbiotech, Taiwan). The two groups were LPS alone (1 μg/mL), KMUP-1 (10 μM) and LPS (1 μg/mL). The gene levels that were significantly modulated by LPS and KMUP-1 were identified.

### 2.8. Intracellular Reactive Oxygen Species (ROS) Measurement

Identification of intracellular ROS was detected by fluorescent stain, a 2′,7′-dichlorodihydrofluorescein diacetate/DCFH-DA (Molecular Probes, Eugene, OR, USA). The cells were seeded in the 96-well plate followed by various concentrations of KMUP-1 pretreatement for 1 h and incubation with LPS for 4 h. Afterward, each well was stained with DCFH-DA (1 μM) for 1 h at 37 °C and immediately washed with PBS. Fluorescence images were captured with a fluorescence microscope (Nikon, TE2000-S, Tokyo, Japan) with 485 nm excitation and 525 nm emission wavelengths.

### 2.9. Animals

Eighteen 6-week-old male Wistar rats, weighing 180–200 g, were purchased from BioLASCO Taiwan Co., Ltd., Taiwan. Rats were randomly divided into three groups, each one containing 6 rats. The rats were housed at 22 ± 2 °C with a relative humidity of 55 ± 10% in a 12-h light–dark cycle with food and sterile tap water available ad libitum. The Animal Care and Use Committee at Kaohsiung Medical University animal center authorized all procedures and protocols (Kaohsiung, Taiwan, IACUC Approval No. 108027).

### 2.10. OA Induction in Rats

Prior to experiment, the rats underwent 1 week acclimatization. To stimulate OA, monosodium iodoacetate (MIA) solution was injected into the intra-articular space of left knee under inhalation of 2% isoflurane anesthesia. The three groups of rats were control group receiving intra-articular saline injection, MIA group receiving intra-articular injection of 4 mg MIA in 25 μL saline, and KMUP-1 + MIA group with oral treatment of 5 mg/kg BW KMUP-1 and MIA injection. KMUP-1 was administered once a day for 7 days in rats after MIA injection. The concentration of KMUP-1 was obtained from our previous study [15]. After the treatment with KMUP-1, no evidence of systemic adverse effects was observed in any study group.

### 2.11. Hindpaw Mechanical Hyperalgesia in Rats

Rats were placed in a wire mesh cage and habituated for 10 min to the environment. An automated dynamic plantar aesthesiometer (Ugo Basile, Varese, Italy) was used to measure the paw withdrawal latency, which was recorded as the time (s) causing a rapid withdrawal of the rat’s left hind leg. Each measurement was repeated three times at intervals of 5 min, and the force evoking reliable withdrawals was 5 g. The medial portion of the left hind paw was placed in the probe of the analgesiometer, and linearly increasing forces were applied to the paw. In the study, hindpaw mechanical hyperalgesia was tested at day 7th, the similar day when the last MIA injection was stimulated into the rats.

### 2.12. Macroscopic and Histopathologic Observations of Cartilage

After the rats were sacrificed at day 14th, the tibia, femur and patella bones were separated and dissected free of muscle. The surface of the femoral groove and femoral condyles were photographed by camera for gross examination. The extracted knee from each group was fixed in 10% formalin. Then the samples were dehydrated in 10% EDTA for 14 days and embedded in paraffin. The 4 μm paraffin sections were stained with Hematoxylin and Eosin stain (H&E) and toluidine blue. The morphological features of the synovium were assessed in H&E-stained slices according to the criteria as previously described [17]. Three sections from each sample were randomly chosen and scored by three blinded observers. To evaluate the cartilage status microscopically, the modified OARSI system scores were applied based on prior studies [18,19]. The possible maximum score of the OARSI system is 18, and the structure was scored on a scale of 0–10. Cellularity was scored on a scale of 0–4. Chondrocyte cloning was scored on a scale of 0–4. In addition, the modified Mankin scores were used to determine groups of different stages of cartilage destruction. Mankin scores 0–2 indicate normal cartilage, 3–5 superficial fibrillation, 6–7 moderate cartilage destruction, 8–10 severe damage of cartilage and over 10 complete loss of cartilage [18]. The slides were evaluated under microscopic observation (Nikon Eclipse TE 2000-S, Tokyo, Japan).

### 2.13. Serum Levels of TNF-α and IL-6

The blood samples were centrifuged at 3000 rpm for 10 min at 4 °C. Supernatants were collected and divided into tubes and stored at −80 °C. The levels of TNF-α and IL-6 in the serum were measured using ELISA assay kits according to the manufacturer’s instructions.

### 2.14. Statistical Analyses

The results were expressed as mean ± standard error of the mean (SEM) from at least three independent experiments. To determine the significance of differences between two groups, Student’s t-test was used. For multiple comparisons, we analyzed with one-way ANOVA. Data was considered statistically significant if *p*-value less than 0.05.

## 3. Results

### 3.1. KMUP-1 Effects on LPS-Induced Cytotoxicity and NO Production

To determine whether KMUP-1 results in cytotoxic effects, we generated the MTT as-say. As shown in Figure 1b, KMUP-1 exhibits no cytotoxic effect in RAW264.7 cells after 24 h KMUP-1 exposure at different concentration levels (1, 5, 10 μM). Effect of 1 h KMUP-1 pretreatment at various concentrations (1, 5, 10 μM) and subsequent 24 h LPS (1 μg/mL) stimulation were defined. The cell viability was significantly increased by pretreatment of KMUP-1 in a dose-dependent manner. Furthermore, NO production was evaluated from cells culture media. As presented in Figure 1c, KMUP-1 significantly repressed the production of NO in LPS-induced cells in the comparison with the LPS group (*p* < 0.01 and *p* < 0.001).

### 3.2. KMUP-1 Inhibits LPS-Induced Inflammatory Cytokines Production

**Figure 2:**
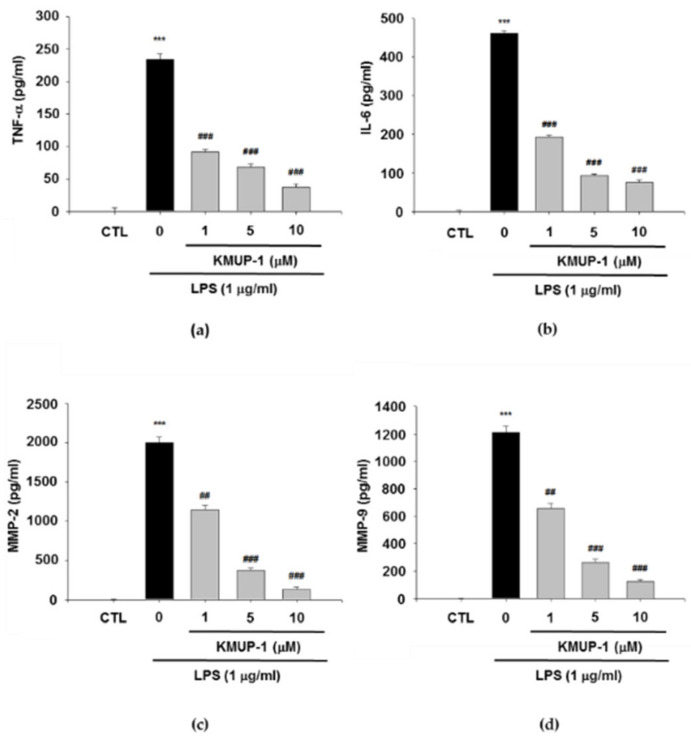
Effects of KMUP-1 on inflammatory cytokines in LPS-induced RAW 264.7 cells. (**a**) Tumor necrosis factor alpha (TNF-α), (**b**) interleukin 6 (IL-6), (**c**) matrix metalloproteinase-2 (MMP-2), and (**d**) matrix metalloproteinase-9 (MMP-9) in the culture media were evaluated by ELISA assay. Values are presented as mean ± SEM, *n* = 3. *** *p* < 0.001 compared to control group. ## *p* < 0.01 and ### *p* < 0.001 compared to LPS group.

The RAW264.7 cells were pretreated with various concentrations of KMUP-1 (1, 5, and 10 μM) for 1 h and further stimulated with 1 μg/mL LPS for 24 h to induce inflammatory cytokines generation. The expressions of TNF-α, IL-6, MMP-2, and MMP-9 were all significantly downregulated by KMUP-1 pretreatment in a dose-dependent manner, respectively (*p* < 0.01 and *p* < 0.001, Figure 2).

### 3.3. KMUP-1 Prevents LPS-Induced Inflammatory Protein Expressions

**Figure 3:**
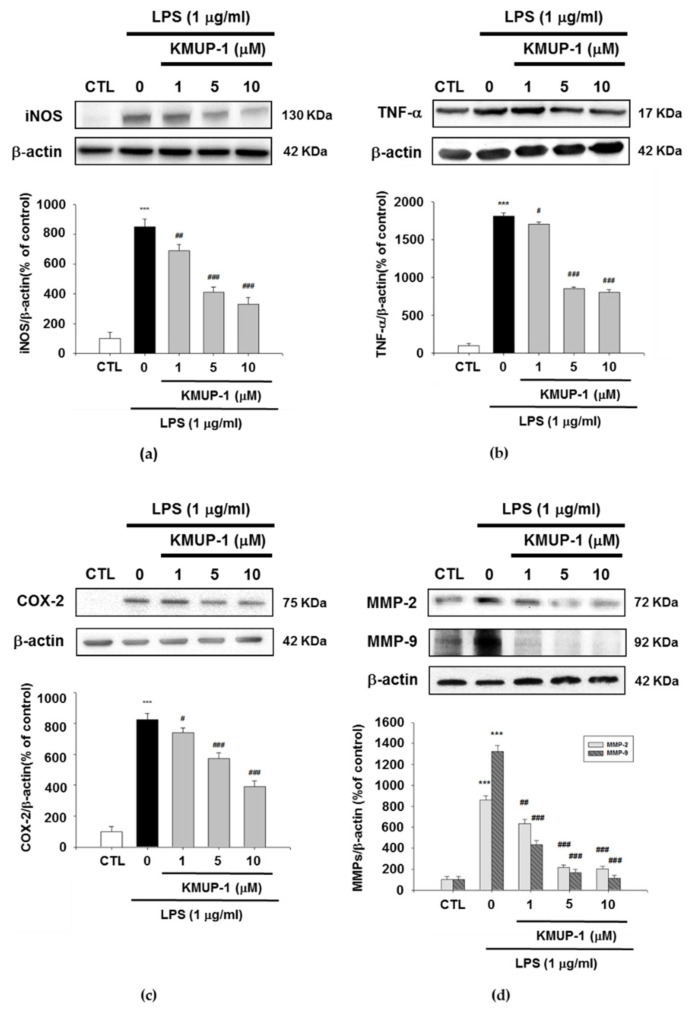
Effect of KMUP-1 on LPS-induced inflammatory reaction proteins expression in RAW 264.7 cells. (**a**) Inducible nitric oxide synthase (iNOS), (**b**) TNF-α, (**c**) cyclooxygenase-2 (COX-2), (**d**) MMP-2, and MMP-9 were detected by Western blot analysis. Values are presented as mean ± SEM, *n* = 3. *** *p* < 0.001 compared to control group. # *p* < 0.05, ## *p* < 0.01 and ### *p* < 0.001 compared to LPS group.

To study whether KMUP-1 blocks either the inflammatory protein expressions of iNOS, TNF-α, COX-2, MMP-2, and MMP-9, we established Western blotting assay. As similar with the result of inflammatory cytokines production, KMUP-1 pretreatment led to the significant decrease of iNOS, TNF-α, COX-2, MMP-2, and MMP-9 expressions, respectively. As shown in Figure 3, the various concentrations of KMUP-1 (1, 5, and 10 μM) reduce LPS-induced inflammatory protein expressions in a dose-dependent manner (*p* < 0.001). Furthermore, as presented in Figure 3a–d, KMUP-1 pretreatment decreases the expression of protein levels of iNOS, TNF-α, COX-2, MMP-2, and MMP-9 as well in LPS-induced inflammation (*p* < 0.001). The KMUP-1 significantly inhibits inflammatory protein expressions in vitro in a dose-dependent manner.

### 3.4. KMUP-1 Downregulates TNF and COX Family Gene Expressions in LPS-Induced Cells

**Figure 4:**
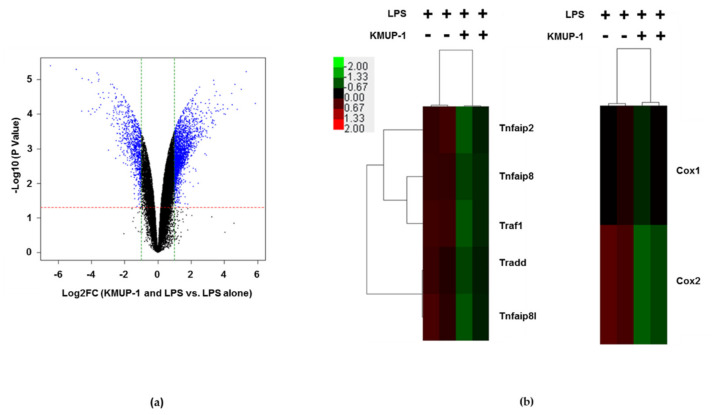
The microarray analysis to determine different expressed transcripts. Volcano plots indicate the differentially expressed transcripts in RAW 264.7 cells pretreatment with 10 μM KMUP-1 for 1 h and/or 1 μg/mL LPS for additional 24 h. The negative log10 transformed *p*-values (*y*-axis) were plotted against the average log2-fold change (*x*-axis) in gene expression. Transcripts with a *p*-value ≤ 0.05 and two-fold up- or downregulated were classified as statistically significant. (**a**) Volcano plot shows LPS-stimulated RAW 264.7 cells compared to LPS + KMUP-1 treatment. Significantly differentially expressed transcripts are showed in blue dotted line, which indicates the -log10 of *p* ≤ 0.05. (**b**) Clustering analysis was performed to visualize the correlations among the replicates and varying sample conditions.

To investigate the influence of KMUP-1 on inflammation induction in LPS-stimulated RAW 264.7 mouse cells, a microarray-based transcriptome analysis was performed. The changes in gene expression by KMUP-1 and LPS is shown on Figure 4. Volcano plots indicate the differentially expressed transcripts in RAW 264.7 cells pretreatment with 10 μM KMUP-1 for 1 h and/or 1 μg/mL LPS for additional 24 h (see Figure 4a). As it should be appropriate for the amount of data and its intrinsic variation achieved from microarray experiments, we used statistical methods to analytically extract relevant information and removed the related uncertainty [20]. To identify the different expressed transcripts, we used volcano plots to simultaneously show the two correlated pieces of information: fold-change and t-statistic. Therefore, scatterplots of the negative log10-transformed *p*-values from the gene-specific *t*-test on the *y*-axis against the log2-fold change on the *x*-axis were prepared. A *p*-value ≤ 0.01 and a fold-change ≥ 3 were used to define significantly modulated gene expression. The profiles of characteristic gene expression are presented in Figure 4a. Among these genes, the heat map showed that *Tnf* and *Cox* family genes were upregulated in LPS-stimulated. In contrast, KMUP-1 treatment significantly decreased their expressions (Figure 4b).

### 3.5. KMUP-1 Inhibits MAPK and NF-κB Signaling Pathways in LPS-Induced Cells

**Figure 5:**
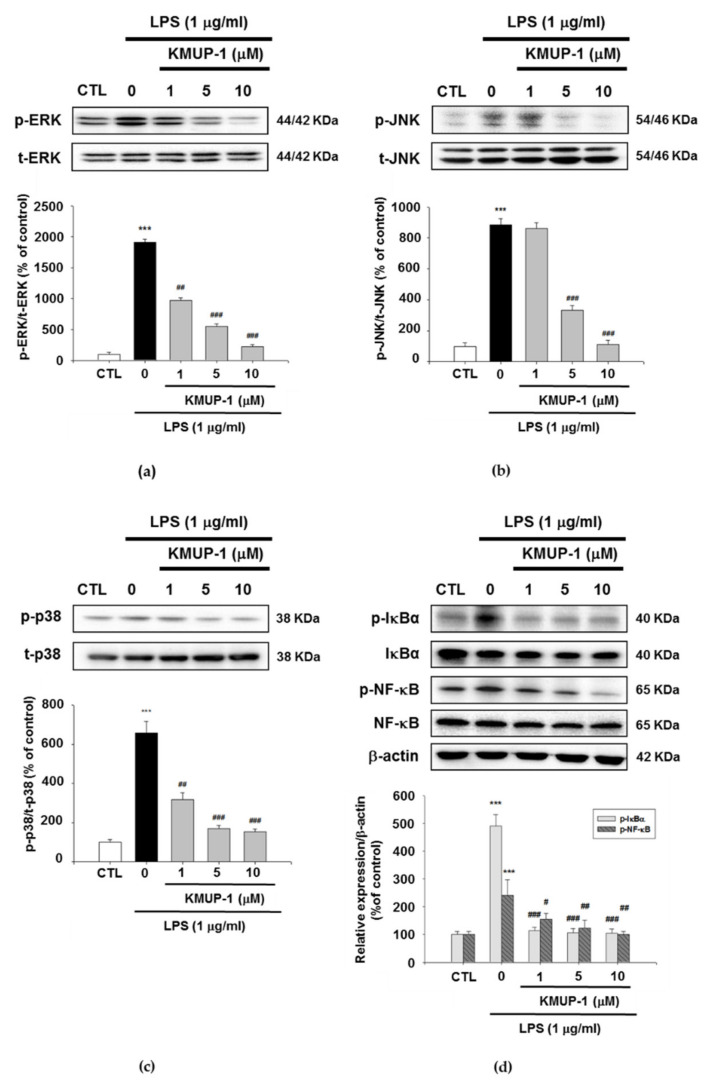
Effects of KMUP-1 on MAPK and NF-κB expressions of LPS-induced RAW 264.7 cells. A representative Western blots demonstrating (**a**) ERK phosphorylation levels, (**b**) JNK protein levels, (**c**) p38 protein levels, and (**d**) IκBα and NF-κB expression levels. Values are presented as mean ± SEM, *n* = 3. *** *p* < 0.001 compared to control group. # *p* < 0.05, ## *p* < 0.01, ### *p* < 0.001 compared to LPS group.

To clarify whether KMUP-1 results in the signaling of mitogen-activated protein kinase (MAPK) and NF-κB pathways, we examined the protein levels through Western blotting. As presented in Figure 5a–c, the LPS stimulation increases phosphorylation levels of ERK, JNK, and p38. Conversely, KMUP-1 pretreatment decreased these protein expressions in a dose-dependent manner. Furthermore, LPS induced the phosphorylation of IκBα and NF-κB. On the contrary, the levels of phosphorylated IκBα and NF-κB expression were reduced by KMUP-1 pretreatment in a dose-dependent manner (Figure 5d). Taken together, these data indicate that KMUP-1 decreases in vitro LPS-induced inflammation through MAPK and NF-κB signaling pathways.

### 3.6. KMUP-1 Suppresses LPS-Induced ROS Production and Restores SIRT1 Level

**Figure 6:**
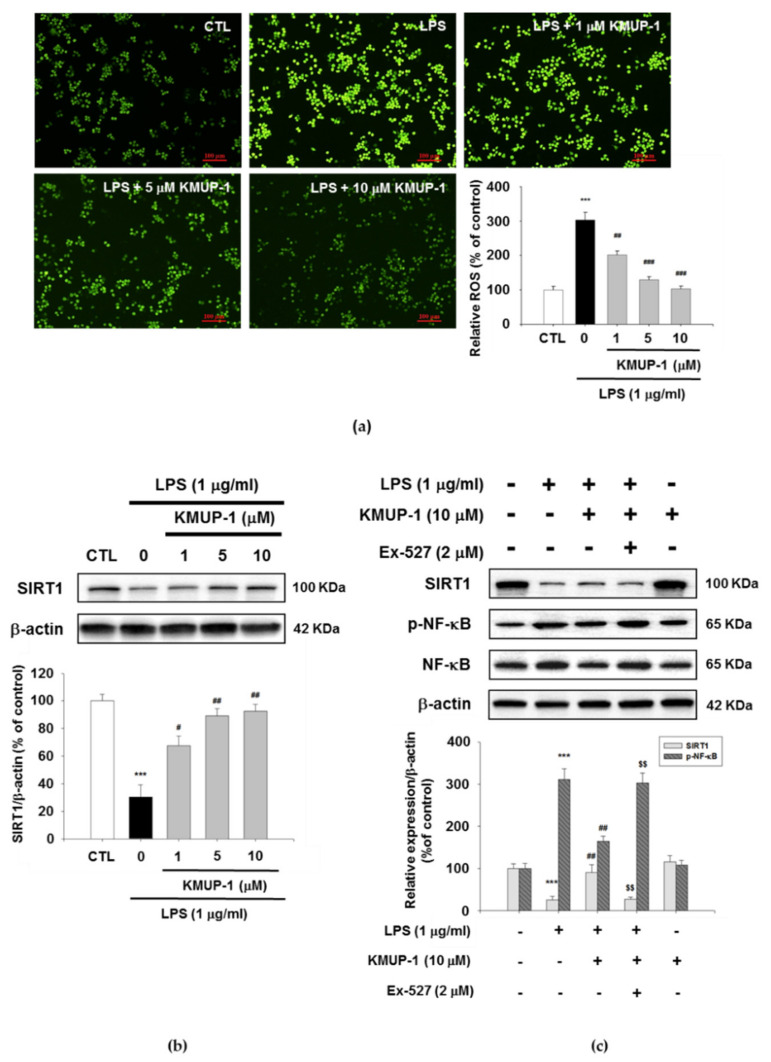
Effects of KMUP-1 on LPS-induced oxidative stress and NF-κB activation regulated by SIRT1 in RAW 264.7 cells. (**a**) The cellular oxidative stress was determined by DCFH-DA staining (100× magnification). A representative Western blots demonstrating (**b**) SIRT1 protein levels (**c**) SIRT1 and NF-κB phosphorylation levels. Values are presented as mean ± SEM, *n* = 3. *** *p* < 0.001 compared to control group. # *p* < 0.05, ## *p* < 0.01, ### *p* < 0.001 compared to LPS group. $$ *p* < 0.01 compared to LPS + KMUP-1 group.

ROS play an important role in the activation of inflammation, thus we examined LPS-induced RAW264.7 cells ROS generation by DCFH-DA fluorescence assay. As shown Figure 6a, the fluorescence intensity showed that KMUP-1 dose-dependently reduces LPS-induced ROS production. Furthermore, the protein level of SIRT1 decreased in LPS stimulation compared to the KMUP-1 pretreatment. In contrast, SIRT1 expression levels were higher in KMUP-1 pretreatment in a dose-dependent manner (Figure 6b). Further, the cells were 1 h treated with 2 μM Ex-527 (the SIRT1 inhibitor) prior to KMUP-1 administration and then stimulated with LPS. As presented in Figure 6c, the protein level of SIRT1 was downregulated by Ex-527 administration in KMUP-1/LPS-treated cells. KMUP-1 inhibits LPS-induced expression level of NF-κB phosphorylation. By contrast, Ex-527 abolished KMUP-1-induced attenuation of NF-κB phosphorylation in KMUP-1/LPS-treated cells. This data indicates that KMUP-1 inhibits in vitro LPS-induced oxidative stress. Particularly, KMUP-1 could result in modulation of NF-κB via SIRT1 restoration in vitro.

### 3.7. KMUP-1 Alleviated Mechanical Hyperalgesia and Serum Inflammatory Cytokines Levels in MIA-Induced OA Rats

**Figure 7:**
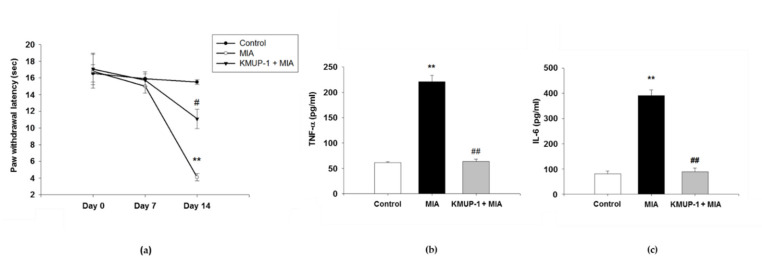
Effects of KMUP-1 on mechanical hyperalgesia and serum levels of inflammatory cytokines in MIA-induced OA rats. (**a**) The paw withdrawal latency (PWL) was measured as described in Section 2. (**b**) The levels of TNF-α and (**c**) IL-6 were evaluated by ELISA assay. Values are presented as mean ± SEM, *n* = 6. ** *p* < 0.01 compared to control group. # *p* < 0.05, ## *p* < 0.01 compared to MIA group.

To mimic the pain in human OA, we established MIA-induced rat model of OA with the alterations in both biochemistry and structure leading to the disease [21]. We further determined the nociceptive response experiment to identify whether KMUP-1 oral pre-treatment has an effect on inflammation-induced pain, a predominant symptom of OA. The mechanical hyperalgesia of paw withdrawal is shown in Figure 7a. In the von Frey hair assessment test, MIA injection significantly reduced the paw withdrawal latency (PWL) in rats as compared to control group on day 14 (*p* < 0.01). The administration of oral 5 mg/kg BW KMUP-1 significantly resulted in the alleviation of mechanical hyperalgesia as compared with the MIA group (*p* < 0.05).

To evaluate whether KMUP-1 has effects on systemic inflammatory parameters, we determined the serum levels of TNF-α and IL-6. As presented in Figure 7b,c, there were significant increases of in TNF-α and IL-6 serum concentrations in MIA-induced OA rats (*p* < 0.01). By contrast, KMUP-1 treatment significantly suppressed these serum levels. It means that KMUP-1 inhibits serum inflammation markers in MIA-induced OA rats (*p* < 0.01).

### 3.8. Protective Effects of KMUP-1 on Articular Cartilage Erosion in MIA-Induced OA Rats

**Figure 8:**
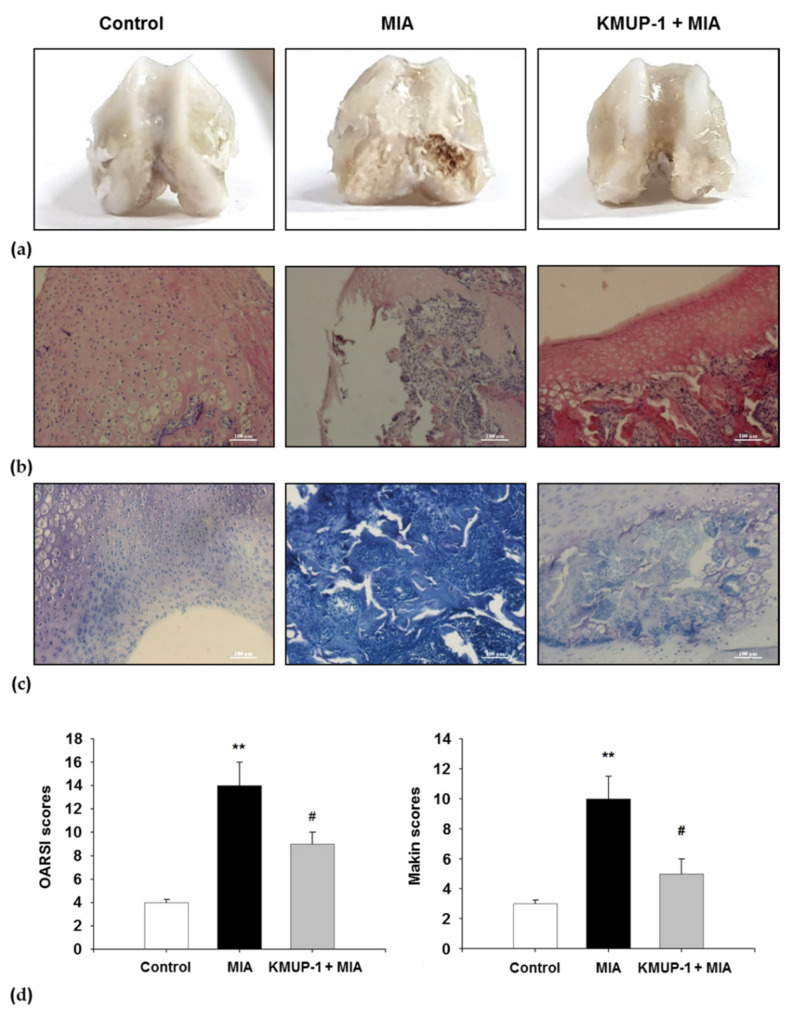
Effect of KMUP-1 on cartilage erosion and chondrocyte disorganization of knee joint in MIA-induced OA rats. (**a**) The gross finding of femoral condyles and femoral groove after MIA injection. (**b**) Hematoxylin and Eosin stain (H&E), and (**c**) toluidine blue (magnification, 100×). (**d**) The joint lesions were microscopically graded according to the modified OARSI and Mankin scores as described in materials and methods. Values are presented as mean ± SEM, *n* = 6. ** *p* < 0.01 compared to control group. # *p* < 0.05 compared to the MIA group.

Further, we evaluated the chondroprotective effect of KMUP-1 through macroscopic observation of the knee joints. We generated three groups for the in vivo study: control, MIA, and MIA + KMUP-1 groups. Figure 8a denotes the macroscopic evaluation of articular cartilage surfaces. On day 7 after intra-articular injection of MIA, oral administration of KMUP-1 resulted to prevent articular cartilage destruction. The area of eroded cartilage surface was significantly reduced in the joints of KMUP-1-treated rats. As shown in Figure 8a, it was found that the injection of MIA resulted in cartilage erosion with large areas of femoral groove and femoral condyles. The staining observation showed smooth articular cartilage and normal cellularity in the control group. In contrast, the joints from MIA-induced OA rats showed narrowing in the joint space along with a marked depletion of proteoglycan. The KMUP-1-treated OA rats significantly reduced these histomorphological cartilage alterations through microscopic analysis (Figure 8b). It was shown in Figure 8c that MIA-induced OA rats were characterized by the involvement of subchondral bone. On 7 days after MIA, injection causes an increase in multinucleated osteoclasts and irregular surface, which was significantly decreased following KMUP-1 treatment. As presented in Figure 8d, the cartilage histological results in MIA group revealed the progression of OA up to the deep zones of the cartilage layers with high OARSI score (*p* < 0.01). On the contrary, it was demonstrated the slower progression of cartilage destruction in KMUP-1-treated group (*p* < 0.05). Similarly, Mankin scores showed significantly lower in KMUP-1-treated group, as compared to the MIA group (*p* < 0.05). In summary, both OARSI and Mankin scores increased with MIA injection. KMUP-1 treatment group showed significantly lower OARSI and Mankin scores, as compared to the MIA group.

## 4. Discussion

The present study demonstrated whether KMUP-1 has potential to inhibit inflammation both through in vitro and in vivo experiments. Various concentrations of KMUP-1 increased cells viability in LPS-induced cytotoxicity in RAW264.7 cells. Furthermore, the inflammatory markers, such as NO, iNOS, TNF-α, COX-2, MMP-2, and MMP-9 were reduced by various concentrations of KMUP-1 pretreatment. Accordingly, KMUP-1 pretreatment led to the downregulation of TNF and COX family genes. Regarding MAPK and NF-κB signaling pathways, pretreatment with KMUP-1 alleviated inflammation through decreased phosphorylation levels of ERK, JNK, and p38. By contrast, KMUP-1 pretreatment inhibited the expression levels of phosphorylated IκBα and NF-κB. Moreover, KMUP-1 resulted in the inhibition of oxidative stress in vitro. Further, SIRT1 protein expression level was downregulated by LPS stimulation, whereas KMUP-1 pretreatment restored its expression in a dose-dependent manner. Moreover, our in vivo study showed comparable findings with in vitro experiments. KMUP-1 led to beneficial effect in MIA-induced OA rats such as macroscopic, microscopic, and functional observations. It is emphasized that KMUP-1 comprehensively protects the dynamic alterations in inflammatory factors and cartilage biomarkers in vivo.

One of the major findings of the research was that KMUP-1 is harmless towards the cultured RAW264.7 cells as our previously study [15], and also in the various cell cultures including cardiomyocytes [22], SH-SY5Y cells [14], GH3 pituitary tumor cells [23], and murine 3T3-L1 pre-adipocytes [24]. Further, these recent studies confirmed that KMUP-1 was essentially free of the toxic compound, and thus it can be suggested that it is probably safe for treatment. According to Liou et al (2013), KMUP-1 exhibited significant inhibition on the osteoclast formation and activation [15]. Similarly, osteoclast formation and activation process during bone metabolism also can be promoted by inflammatory signals. Therefore, this study offered some insight to define the protective effects of KMUP-1 in LPS-induced RAW264.7 cells. Moreover, we observed that KMUP-1 administration could suppress LPS-induced cytotoxicity and nitrite production. It means that the administration of KMUP-1 results in decreased cell cytotoxicity as well as lessened nitrite released in LPS-induced RAW264.7 cells.

In the pathogenesis of OA, inflammatory mediators, mechanical stimulation, oxidative stress, and cellular damage collude in the function and viability of chondrocytes, reprogramming them to undergo hypertrophic differentiation and initial aging, leading them to be responsive to the effects of proinflammatory and procatabolic mediators [4]. The fundamental pathophysiological pathways encompassed in OA involve some the typical assumes, so called proinflammatory TNF-α and interleukins (IL-1β, IL-6, and IL-8), and procatabolic mediators through their signaling pathways and the well-defined effects of MAPK and NF-κB signaling responses in addition to reprogramming are ‘switching’ pathways in transcriptional networks [25].

The most common forms of arthritis and the major cause of disability is OA, but there is no approved drug to stop or slow the disease progression [26]. More recent evidence reveals that inflammation plays a pivotal role in the pathology of OA, which recommends that a suitable therapeutic strategy for OA is targeting inflammation [27,28]. In previous studies, xanthine-based derivative KMUP-1 exhibits its bioactive effects including anti-inflammatory [12], cardio-protection [13], antioxidative stress, neuroprotective and presenting bone metabolism function [15,16]. Dai et al. (2014) demonstrated that KMUP-1 attenuated inflammation in the sciatic nerve tissues through lessening proinflammatory cytokines (TNF-α and IL-1β), decreased the expression levels of COX-2, iNOS, nNOS, MAPKs, p-IκB, and p-NF-κB. Further, their findings confirmed the pathway of KMUP-1 was through the inhibition of NF-κB upregulation and the MAPKs [29]. In this study, we determined whether KMUP-1 could suppress LPS-induced production of inflammatory mediators, NF-κB activation, and ROS production in RAW264.7 cells. Additionally, KMUP-1 decreased the progression of inflammation-induced cartilage destruction in MIA-induced rat OA. An implication of this is the possibility that inflammatory cytokines have pivotal roles in the progression of OA [2]. LPS, a main component of the outer membrane of Gram-negative bacterial factor that has the ability to induce the expression of a variety of proinflammatory cytokines, is commonly used to mimic inflammation in RAW264.7 cells in in vitro culturing systems [30]. Furthermore, a class of proteinases, MMPs, MMP-1, MMP-3 and MMP-13 regulate various functions including extracellular matrix (ECM) degradation in OA and these MMPs have been reported to play an important role in the progression of OA [31]. In recent years, there has been an increasing amount of literature on the characterization of increased production of MMP-1 and MMP-13 through in vitro [32,33] and in vivo [34] studies. However, recent studies showed that MMP-2 and MMP-9 were activated in patients with OA [35,36]. Lipari and Garbino (2013) showed the high expression levels of MMP-2 and MMP-9 in OA patients compared with controls [35]. Zeng et al. (2015) indicated that the protein levels of MMP-1 and MMP-2 were higher in Asian patients with OA than in controls [36]. Therefore, our study was conducted to determine whether KMUP-1 has the potential to block the expressions of MMP-2 and MMP-9 in the in vitro experiments on RAW264.7 cells. The evidence from this study suggests that KMUP-1 may exert anti-inflammatory effects by suppressing the important inflammatory mediators induced by LPS in vitro.

There has been a significant increase in new research that aims to decipher the mechanisms of sirtuin function and their roles in pathology of OA [9,37]. The majority of the work carried out to date has focused on SIRT1 which is essential for maintaining cartilage homeostasis. In this study, we demonstrated that KMUP-1 has beneficial effects on articular cartilage anabolism by encouraging cell survival, especially under LPS-induced stress conditions, which may provide a mechanism supporting the therapeutic potential intervention in OA.

Additionally, stimulation of RAW264.7 cells by LPS induces phosphorylation and the activation of ERK, JNK, and p38. Similarly, in our previous study we proved that KMUP-1 inhibited RANKL-induced phosphorylation of MAPK in RAW264.7 cells. Osteoclastogenesis is the process during bone metabolism which can also be activated by inflammatory pathways [38]. It has been demonstrated that MAPK, including ERK, p38, and JNK, play a crucial role in the IL-1 regulation of MMPs expression and subsequent cartilage destruction. MAPK also played an important role in the regulation of inflammatory mediators production [39,40], and could be specifically activated downstream to overproduce COX-2, iNOS, TNF-α, IL-6, MMP-2, and MMP-9 [36]. Thus, the anti-inflammatory agents that could prevent phosphorylation of MAPK may be a beneficial treatment for OA in the model of osteoarthritis chondrocytes [39,40].

More recent evidence suggests the important role of signaling pathways through NF-κB and MAPK in the regulation of inflammatory mediators involved in the pathogenesis of OA [31,41]. As normal, NF-κB forms as an inactive transcription factor in cytoplasm which is associated with IκB, an inhibitory protein. Upon stimulation by LPS activates the IκB kinase (IKK) complex and then leads to the phosphorylation and degradation of IκB, leading to the nuclear translocation of NF-κB to regulate the expression of inflammatory mediators [42,43]. Additionally, the NF-κB constitutes a family of transcription factors that are stimulated by proinflammatory cytokines, chemokines, stress-related factors ECM degradation products, and by LPS. The activated NF-κB molecules trigger the expression of an array of genes leading to promote major proinflammatory expression and destructive mediators of OA, increase matrix-degrading enzyme production, thereby contributing to OA onset and development [3,44,45,46]. Therefore, we investigated whether the anti-inflammatory effects of KMUP-1 were through NF-κB signaling pathways. Our data indicated that the anti-inflammatory and antioxidant activity properties of KMUP-1 resulted from the inhibited phosphorylation of IκB and NF-κB induced by LPS.

There has been a significant increase in new research that aims to decipher the mechanisms of sirtuin function and their roles in pathology of OA [9,37]. The majority of the work carried out to date has focused on SIRT1 which is essential for maintaining cartilage homeostasis. In this study, we demonstrated that KMUP-1 has potential beneficial effects on articular cartilage anabolism by alleviating inflammation in vivo, which may provide a mechanism supporting the therapeutic potential intervention in OA. Furthermore, cells treated with SIRT1 inhibitor significantly attenuated the repressive effect of KMUP-1 on LPS-induced NF-κB activation. SIRT1 inhibits NF-κB signaling directly by deacetylated the p65 subunit of NF-κB complex, the inhibition of SIRT1 disrupts oxidative energy metabolism and stimulates the NF-κB-induced inflammatory responses present in many chronic metabolic and age-related diseases [10,11]. Therefore, our data showed that KMUP-1 effects in the inhibition of IκB/NF-κB signaling pathways was due, in part, to mediation by SIRT1 in vitro.

The cartilage degradation is a major mechanism involved in the progression of OA. Pharmacological treatments for OA are to alleviate pain and to improve function. The treatment should be, therefore, personalized since not every patient will benefit from a specific treatment. The OA paradigm has shifted from degenerative joint disease to inflammatory joint disease and increasing understanding about the metabolic role in OA has led to new opportunities for OA treatment. Our study is first to show the anti-inflammatory effect of KMUP-1 pretreatment in LPS-induced inflammation in RAW264.7 mouse macrophages. Furthermore, our findings demonstrated that KMUP-1 decreased LPS-induced activation of MAPK and NF-κB signaling pathways. Particularly, the SIRT1 expression level was restored in the LPS-induced RAW264.7 cells. Additionally, our study showed that KMUP-1 has a potential to inhibit mechanical-stimulated pain, inflammation and cartilage destruction in the OA rat model. Taken together, we hypothesized that these findings indicate that KMUP-1 may serve as a potential anti-inflammatory agent in the treatment of OA.

## 5. Conclusions

In this study, we provided evidence that KMUP-1 inhibits cytotoxicity, inflammatory cytokines production, oxidative stress, MAPK and IκB/NF-κB activation in LPS-induced RAW264.7 cells. Moreover, KMUP-1 led to the restoration of the expression level of SIRT1, in part due to the suppression of IκB/NF-κB phosphorylation in vitro. KMUP-1 also alleviated the hyperalgesia and cartilage destruction in the OA rat model. Our results demonstrated that KMUP-1 particularly prevents MIA-induced OA symptoms via mechanisms involving its anti-inflammatory effect. Further studies would be beneficial to explore many molecular cascades involved in inflammatory response syndrome extended clinical studies. KMUP-1 may be a promising therapeutic agent for OA and management of its associated symptoms.

## Data Availability

Data presented in this study can be requested from the corresponding author, Jwu-Lai Yeh.

## References

[1] Berenbaum F. (2013). Osteoarthritis as an inflammatory disease (osteoarthritis is not osteoarthrosis!). Osteoarthr. Cartil..

[2] Goldring M.B., Otero M. (2011). Inflammation in osteoarthritis. Curr. Opin. Rheumatol..

[3] Kapoor M., Martel-Pelletier J., Lajeunesse D., Pelletier J.-P., Fahmi H. (2011). Role of proinflammatory cytokines in the pathophysiology of osteoarthritis. Nat. Rev. Rheumatol..

[4] Mobasheri A., Batt M. (2016). An update on the pathophysiology of osteoarthritis. Ann. Phys. Rehabil. Med..

[5] Castrogiovanni P., Di Rosa M., Ravalli S., Castorina A., Guglielmino C., Imbesi R., Vecchio M., Drago F., Szychlinska M.A., Musumeci G. (2019). Moderate physical activity as a prevention method for knee osteoarthritis and the role of synoviocytes as biological key. Int. J. Mol. Sci..

[6] Szychlinska M.A., Castrogiovanni P., Nsir H., Di Rosa M., Guglielmino C., Parenti R., Calabrese G., Pricoco E., Salvatorelli L., Magro G. (2017). Engineered cartilage regeneration from adipose tissue derived-mesenchymal stem cells: A morphomolecular study on osteoblast, chondrocyte and apoptosis evaluation. Exp. Cell Res..

[7] Guarente L. (2011). Sirtuins, aging, and medicine. N. Engl. J. Med..

[8] Morris B.J. (2013). Seven sirtuins for seven deadly diseases of aging. Free Radic. Biol. Med..

[9] Dvir-Ginzberg M., Mobasheri A., Kumar A. (2016). The role of sirtuins in cartilage homeostasis and osteoarthritis. Curr. Rheumatol. Rep..

[10] Kauppinen A., Suuronen T., Ojala J., Kaarniranta K., Salminen A. (2013). Antagonistic crosstalk between NF-κB and SIRT1 in the regulation of inflammation and metabolic disorders. Cell Signal..

[11] Yeung F., Hoberg J.E., Ramsey C.S., Keller M.D., Jones D.R., Frye R.A., Mayo M.W. (2004). Modulation of NF-κB-dependent transcription and cell survival by the SIRT1 deacetylase. EMBO J..

[12] Wu B.-N., Chen C.-W., Liou S.-F., Yeh J.-L., Chung H.-H., Chen J. (2006). Inhibition of proinflammatory tumor necrosis factor-α-induced inducible nitric-oxide synthase by xanthine-based 7-[2-[4-(2-chlorobenzene) piperazinyl] ethyl]-1, 3-dimethylxanthine (KMUP-1) and 7-[2-[4-(4-nitrobenzene) piperazinyl] ethyl]-1, 3-dimethylxanthine (KMUP-3) in rat trachea: The involvement of soluble guanylate cyclase and protein kinase G. Mol. Pharmacol..

[13] Yeh J.L., Hsu J.H., Wu P.J., Liou S.F., Liu C.P., Chen I.J., Wu B.N., Dai Z.K., Wu J.R. (2010). KMUP-1 attenuates isoprenaline-induced cardiac hypertrophy in rats through NO/cGMP/PKG and ERK1/2/calcineurin A pathways. Br. J. Pharmacol..

[14] Hsu Y.-Y., Liu C.-M., Tsai H.-H., Jong Y.-J., Chen J., Lo Y.-C. (2010). KMUP-1 attenuates serum deprivation-induced neurotoxicity in SH-SY5Y cells: Roles of PKG, PI3K/Akt and Bcl-2/Bax pathways. Toxicology.

[15] Liou S.-F., Hsu J.-H., Lin I.-L., Ho M.-L., Hsu P.-C., Chen L.-W., Chen J., Yeh J.-L. (2013). KMUP-1 suppresses RANKL-induced osteoclastogenesis and prevents ovariectomy-induced bone loss: Roles of MAPKs, Akt, NF-κB and calcium/calcineurin/NFATc1 pathways. PLoS ONE.

[16] Liou S.F., Hsu J.H., Chu H.C., Lin H.H., Chen I.J., Yeh J.L. (2015). KMUP-1 promotes osteoblast differentiation through cAMP and cGMP pathways and signaling of BMP-2/Smad1/5/8 and Wnt/β-catenin. J. Cell. Physiol..

[17] Liu H., Ding J., Wang J., Wang Y., Yang M., Zhang Y., Chang F., Chen X. (2015). Remission of collagen-induced arthritis through combination therapy of microfracture and transplantation of thermogel-encapsulated bone marrow mesenchymal stem cells. PLoS ONE.

[18] Pester J.K., Stumpfe S., Steinert S., Marintschev I., Aurich M., Hofmann G.O. (2013). BMP-2 shows characteristic extracellular patterns in osteoarthritic cartilage: A preliminary report. GMS Interdiscip. Plast. Reconstr. Surg. DGPW.

[19] Waldstein W., Perino G., Gilbert S.L., Maher S.A., Windhager R., Boettner F. (2016). OARSI osteoarthritis cartilage histopathology assessment system: A biomechanical evaluation in the human knee. J. Orthop. Res..

[20] Cui X., Churchill G.A. (2003). Statistical tests for differential expression in cDNA microarray experiments. Genome Biol..

[21] Jeong J.-H., Moon S.-J., Jhun J.-Y., Yang E.-J., Cho M.-L., Min J.-K. (2015). Eupatilin exerts antinociceptive and chondroprotective properties in a rat model of osteoarthritis by downregulating oxidative damage and catabolic activity in chondrocytes. PLoS ONE.

[22] Lee M.-L., Sulistyowati E., Hsu J.-H., Huang B.-Y., Dai Z.-K., Wu B.-N., Chao Y.-Y., Yeh J.-L. (2019). KMUP-1 ameliorates ischemia-induced cardiomyocyte apoptosis through the NO–cGMP–MAPK signaling pathways. Molecules.

[23] Lo Y.C., Tseng Y.T., Liu C.M., Wu B.N., Wu S.N. (2015). Actions of KMUP-1, a xanthine and piperazine derivative, on voltage-gated Na+ and Ca2+-activated K+ currents in GH3 pituitary tumour cells. Br. J. Pharmacol..

[24] Liu C.-P., Chau P.-C., Chang C.-T., An L.-M., Yeh J.-L., Chen I.-J., Wu B.-N. (2018). KMUP-1, a GPCR modulator, attenuates triglyceride accumulation involved MAPKs/Akt/PPARγ and PKA/PKG/HSL signaling in 3T3-L1 preadipocytes. Molecules.

[25] Barreto G., Manninen M., Eklund K.K. (2020). Osteoarthritis and Toll-like receptors: When innate immunity meets chondrocyte apoptosis. Biology.

[26] Ohtake P.J., Lee A.C., Scott J.C., Hinman R.S., Ali N.A., Hinkson C.R., Needham D.M., Shutter L., Smith-Gabai H., Spires M.C. (2018). Physical impairments associated with post–intensive care syndrome: Systematic review based on the world health organization’s international classification of functioning, disability and health framework. Phys. Ther..

[27] Philp A.M., Davis E.T., Jones S.W. (2017). Developing anti-inflammatory therapeutics for patients with osteoarthritis. Rheumatology.

[28] Liu-Bryan R. (2015). Inflammation and intracellular metabolism: New targets in OA. Osteoarthr. Cartil..

[29] Dai Z.-K., Lin T.-C., Liou J.-C., Cheng K.-I., Chen J.-Y., Chu L.-W., Chen I.-J., Wu B.-N. (2014). Xanthine derivative KMUP-1 reduces inflammation and hyperalgesia in a bilateral chronic constriction injury model by suppressing MAPK and NFκB activation. Mol. Pharm.

[30] Kong L., Smith W., Hao D. (2019). Overview of RAW264. 7 for osteoclastogensis study: Phenotype and stimuli. J. Cell Mol. Med..

[31] Feng Z., Li X., Lin J., Zheng W., Hu Z., Xuan J., Ni W., Pan X. (2017). Oleuropein inhibits the IL-1β-induced expression of inflammatory mediators by suppressing the activation of NF-κB and MAPKs in human osteoarthritis chondrocytes. Food Funct..

[32] Nakamura H., Tanaka M., Masuko-Hongo K., Yudoh K., Kato T., Beppu M., Nishioka K. (2006). Enhanced production of MMP-1, MMP-3, MMP-13, and RANTES by interaction of chondrocytes with autologous T cells. Rheumatol. Int..

[33] Klatt A.R., Paul-Klausch B., Klinger G., Kühn G., Renno J.H., Banerjee M., Malchau G., Wielckens K. (2009). A critical role for collagen II in cartilage matrix degradation: Collagen II induces pro-inflammatory cytokines and MMPs in primary human chondrocytes. J. Orthop. Res..

[34] Bau B., Gebhard P.M., Haag J., Knorr T., Bartnik E., Aigner T. (2002). Relative messenger RNA expression profiling of collagenases and aggrecanases in human articular chondrocytes in vivo and in vitro. Arthritis Rheum..

[35] Lipari L., Gerbino A. (2013). Expression of gelatinases (MMP-2, MMP-9) in human articular cartilage. Int. J. Immunopathol. Pharmacol..

[36] Zeng G., Chen A., Li W., Song J., Gao C. (2015). High MMP-1, MMP-2, and MMP-9 protein levels in osteoarthritis. Genet. Mol. Res..

[37] Matsushita T., Sasaki H., Takayama K., Ishida K., Matsumoto T., Kubo S., Matsuzaki T., Nishida K., Kurosaka M., Kuroda R. (2013). The overexpression of SIRT1 inhibited osteoarthritic gene expression changes induced by interleukin-1β in human chondrocytes. J. Orthop. Res..

[38] Tanaka Y., Nakayamada S., Okada Y. (2005). Osteoblasts and osteoclasts in bone remodeling and inflammation. Curr. Drug Targets Inflamm. Allergy.

[39] Thalhamer T., McGrath M., Harnett M. (2008). MAPKs and their relevance to arthritis and inflammation. Rheumatology.

[40] Feng Z., Zheng W., Li X., Lin J., Xie C., Li H., Cheng L., Wu A., Ni W. (2017). Cryptotanshinone protects against IL-1β-induced inflammation in human osteoarthritis chondrocytes and ameliorates the progression of osteoarthritis in mice. Int. Immunopharmacol..

[41] Ma Z., Piao T., Wang Y., Liu J. (2015). Astragalin inhibits IL-1β-induced inflammatory mediators production in human osteoarthritis chondrocyte by inhibiting NF-κB and MAPK activation. Int. Immunopharmacol..

[42] Olivotto E., Otero M., Marcu K.B., Goldring M.B. (2015). Pathophysiology of osteoarthritis: Canonical NF-κB/IKKβ-dependent and kinase-independent effects of IKKα in cartilage degradation and chondrocyte differentiation. RMD Open.

[43] Lu Y.-C., Yeh W.-C., Ohashi P.S. (2008). LPS/TLR4 signal transduction pathway. Cytokine.

[44] Choi M.-C., Jo J., Park J., Kang H.K., Park Y. (2019). NF-κB signaling pathways in osteoarthritic cartilage destruction. Cells.

[45] Rigoglou S., Papavassiliou A.G. (2013). The NF-κB signalling pathway in osteoarthritis. Int. J. Biochem. Cell Biol..

[46] Pulai J.I., Chen H., Im H.-J., Kumar S., Hanning C., Hegde P.S., Loeser R.F. (2005). NF-κB mediates the stimulation of cytokine and chemokine expression by human articular chondrocytes in response to fibronectin fragments. J. Immunol..

